# Putting the Luck Back Into Moral Luck

**DOI:** 10.1111/misp.12104

**Published:** 2019-07-11

**Authors:** Neil Levy

In philosophy, the more constraints we bring to the consideration of a problem the better. This is particularly the case in normative domains, in which the invocation of intuitions may be inevitable. Since our intuitions are never entirely consistent even in a single individual, and different individuals may have somewhat different intuitions (as recent work on experimental philosophy has been at pains to demonstrate), every account of a philosophically interesting explanandum is revisionary to some extent. Every account has costs, in terms of conflict with intuitions, and the narrower the set of considerations we bring to choosing between them, the harder it will be to engage in principled choice between rivals. Broader sets of considerations are often essential to showing that one (somewhat counterintuitive) account is better than another.

Debates over moral luck concern concepts that are largely (though not exclusively) normative. We ought therefore to expect that the narrower the range of considerations that we bring to bear on this debate, the harder it will be to give genuinely principled reasons for preferring one account over others. This fact gives us a strong reason for preferring an account that is sensitive to the full range of considerations that can be brought to bear. Yet the majority of philosophers who have thought about moral luck ignore—or argue for ignoring—the “luck” component of “moral luck.” They substitute for “luck” a lack of control condition, sometimes even while acknowledging that “luck” cannot be captured by such a condition.1Hartman (2017), who accepts this view, calls it the Standard View, and attributes it to Nagel (1979) and Williams (1981)—the founders of the recent debate—as well as Thomson (1989), Greco (1995), Card (1996), Domsky (2004) and Hanna (2014), among others. In this paper, I argue that it is a mistake to substitute “lack of control” for “luck.” If we make that substitution, we will both fail to understand the phenomenon of moral luck, narrowly construed, and we will cut ourselves off from the many ways in which considerations outside that debate bear on and help constrain our thinking and helpfully narrow the options we confront in theory building. We neglect the enormous work on the psychology of luck (and the connections between that work and work in evolutionary theory on the development of psychological dispositions). We neglect the range of luck problems that arise within philosophy itself, in epistemology, in political philosophy, and in the free will debate, none of which can be adequately understood without an account of luck.

Neglect the broader set of issues and we also obscure the most important stakes of the moral luck debate. Stipulating that we will understand the “luck” in “moral luck” as equivalent to lack of control will not prevent a two‐way interaction between our thoughts about moral luck and the ways in which we think about luck and desert in other domains. Our thoughts about moral luck are influenced by our broader views about luck, and the account of moral luck we settle on will have effects on how we think about luck in other domains. Luck and desert are *central* to our politics. Luck egalitarianism is an attractive account of distributive justice because it reflects the extent to which ordinary people tend to think of luck and desert as anticorrelated. We cannot, and should not, insulate the moral luck debate from these broader concerns. Far from succeeding, I suspect that attempts to insulate moral luck from wider concerns about justice will bring about a covert influence of the former on the latter, which is all the stronger for being uncontrolled.

In the first half of this paper, I will defend my account of luck, which combines lack of control with a modal condition and a significance condition, against those who would substitute a lack of control condition alone. In the second half, I turn to broader considerations, to draw out the issues that are at stake in debates over luck. There is less in the way of traditional philosophical argument in this second half. I am more concerned here with drawing linkages between the moral luck debate and broader questions; linkages opened up when we focus on the role that luck—itself—plays in all of them.

## 1 Against the Lack of Control Condition

In *Hard Luck* (Levy 2011), I defended an account of luck that (as I acknowledged) drew heavily on the work of E. J. Coffman (2007) and—especially—Duncan Pritchard (2005). According to that account, lack of control is a necessary but not sufficient condition for an event or state of affairs to be lucky. An event is lucky for an agent if she lacks direct control over its occurrence, it is significant for her and it is modally fragile, which is to say that it occurs in the actual world but fails to occur in a large enough proportion of nearby possible worlds. A world is nearby if it differs from the actual world in at most trivial respects. The proportion of possible worlds that are large enough for an event to count as lucky is a function of the significance of the event: the more significant it is, the lower the proportion. In addition to this account, I introduced an account of what I called *nonchancy* luck: luck in the absence of modal fragility. States of affairs are nonchancy lucky when they are significant for the agent, she lacks direct control over them, and states of affairs like that are relatively uncommon across a relevant reference group. Thus one might be unlucky to be born with a severe disability. Arguably, the disability might not be modally fragile, because there is no world in which the agent herself lacked that disability. But because being disabled in that kind of way is relatively uncommon among her contemporaries, it satisfies the analog of the fragility condition.

*Hard Luck* focused on the free will debate, not the moral luck debate. Nevertheless, I contend that the account of luck developed there is the appropriate one for the moral luck debate, too. What Thomas Nagel called resultant luck—luck in how events turn out—is a kind of chancy luck. Constitutive luck (luck in the traits one happens to have) and circumstantial luck (luck in the circumstances in which one acts) are sometimes chancy and sometimes nonchancy luck. Of the four kinds of luck Nagel identified, only causal luck is not adequately captured by the account. But since I strongly suspect that the problem of causal luck is misnamed—it is simply the question whether free will is compatible with causal determinism, and has nothing especially to do with luck—I am not worried by this limitation.

In his recent *In Defense of Moral Luck*, Robert J. Hartman (2017) has produced the most sustained and powerful defense of the claim that we should substitute “lack of control” for “luck” for the purposes of the moral luck debate. Hartman concedes that “lack of control” is not equivalent to “luck”—there are events that are out of control and yet are not lucky for us, like the rising of the sun—but holds that that just shows that the moral luck debate does not turn on luck. Hartman contends that the puzzle of moral luck arises from lack of control, rather than luck itself. The puzzling cases, after all, compare otherwise identical agents who exercise identical degrees of control in relevantly similar circumstances, but who cause very different amounts of harm. Since the degree of control is held fixed across these cases, Hartman claims that the moral luck debate “is about not luck per se but a tension in our ordinary thinking about moral responsibility” (24), and this tension is captured by a lack of control condition. I shall argue that the lack of control condition cannot do all the work we require of it in the moral luck debate.

The paradigm moral luck case is surely the reckless driver. Suppose that Frog and Toad both drive at unsafe speeds through the streets of Tilsbury. Frog careens around a blind corner and collects Badger's bike, with the result that Badger spends 3 months in traction. Toad is luckier; his car whizzes by Mole with inches to spare and no harm is done. The common intuition, of course, is that while nothing (in terms of character, control, or foresight) distinguishes the amphibians, Frog is more blameworthy than Toad. Prima facie, of course, Hartman's lack of control condition can do the work in generating the apparent puzzle that arises from this case: given the centrality of control to moral responsibility and blameworthiness, how can agents who exercise identical degrees of control be due different amounts of blame? But now consider this third case. Newt drives her car recklessly down the Tilsbury High Street on the morning of the cheese appreciation parade. Poor Hedgehog is sent crashing into the parade float by Newt's car, and is lucky to escape with broken bones. Now whatever else we should think of Newt's behavior, it should be apparent that she was not (un)lucky to injure someone. If anything, our intuition is just the reverse: we might say she was lucky that things weren't much worse. She did, after all, drive recklessly in the vicinity of a large crowd of people.

Newt did not control the fact that she hit Hedgehog. She didn't even control the fact that she hit someone or other. She lacked control just as much as did Frog and Toad. But her case is crucially different from theirs: it does not feature moral luck (or at least it does not feature the resultant luck that features in theirs). She was not lucky to hit someone, since hitting someone is the expected upshot of driving like that in conditions like those. A lack of control condition is therefore not adequate to capture differences between cases. Adding a modal condition, however, allows us to understand her case, as well as theirs. The outcome of Frog's driving is modally fragile: in a large proportion of nearby possible worlds he does not hit anyone. That's at least part of the reason that he's unlucky to hit Badger. But (individuated in a way that captures its moral significance: *that someone was injured*), the outcome of Newt's driving was not modally fragile. If you drive recklessly down the Tilsbury High Street the day of the cheese appreciation parade, you are very likely to hit someone. I doubt we can dispense with this modal condition, even for the purposes of understanding paradigm resultant luck cases like this.

It is worth adding that the lack of control condition risks begging the question against certain views of moral responsibility. Consider the family of views according to which we are directly responsible for actions (and omissions) that are expressions of our real selves. These accounts are motivated, in important part, by the difficulty in squaring our intuitions about moral responsibility with the requirements of a control condition. Sometimes agents seem praiseworthy or blameworthy in cases in which they lacked control. Consider omissions cases (Smith 2005). Many people have the intuition, for example, that if a close friend fails to wish them a happy birthday, they are due some blame, and this intuition doesn't dissipate if they discover that the friend simply forgot. But we do not control our forgetting (in any but the most recherché of cases). Real self theorists hold that we may be responsible in cases like this, despite the lack of control, if our omission is an expression of who we genuinely are. It is easy to see how this kind of view provokes trouble for the lack of control condition as sufficient for moral luck.

When Hedgehog gently remonstrates with Owl for his not phoning her, she will not accept as an excuse that he forgot. Hedgehog may, however, be alive to the problem of moral luck, and disposed to excuse or mitigate agents who are unlucky, “But you weren't unlucky,” she insists to Owl. If Owl's omission was indeed an expression of the kind of person he is—if he simply doesn't care sufficiently for her and her welfare, or is characteristically neglectful, say—then he might be responsible for it, *despite* his lack of control. We can best capture why he cannot avail himself of luck as an excuse by reference to the modal condition: in a large proportion of nearby possible worlds, Owl would have omitted to phone Hedgehog just as he did in the actual world (that's the kind of bird he is). If we are not to beg the question against real self theorists, we need an account of luck that enables them to state why the luck excuse is not available in cases like this. Of course, at the end of the day we might want to conclude that a luck excuse is not available to anyone at all, but it's not yet the end of the day. We are setting up the problem, not solving it. We want to capture the apparent paradox of moral luck, as Hartman suggests, and for that a lack of control condition is inadequate.

We cannot, I conclude, capture the apparent paradox that arises from moral luck with an absence of control condition alone. But does adding a modal condition help? As well as arguing that absence of control is all we need, Hartman also argues against what he calls the Augmentation View; a view that, like mine, adds extra conditions to lack of control. He claims that such accounts don't generate the paradox of moral luck at all. He gives the following example to make the point. Jim tells a lie, at the very moment a bolt of lightning strikes nearby. In nearby possible worlds, though, Jim would have been struck by the lightning, and therefore would not have lied. According to the modal account, Hartman concludes, Jim is lucky to lie, and therefore there is a prima facie excuse available to him. Hartman (rightly) objects that we do not think that even a prima facie excuse is available to him, in virtue of his not being hit by lightning, and concludes that the lack of control condition can make sense of our intuition here (26).

This counterexample seems to me to fail. Actually, it fails twice over, but the first problem is easily remediable. The first problem is that as the case is presented it is not plausible that Jim is lucky to avoid being hit by lightning. Lightning is a relatively rare event, and is relatively randomly distributed. Granted there are circumstances in which a person serves as an attractor—they are the highest point around, say. But in those cases they are invariably struck, if the lightning is close. I put this objection aside, however, because I think the case can easily be modified so the liar is lucky to be in a position to lie. That brings us immediately to the second worry, however: what is at issue is, precisely, *being in a position to lie* (to exercise control, as Hartman says) and not *lying*. Jim is lucky to be in such a position, but that does not entail he is lucky to lie. The lightning is irrelevant to what we might call the actual sequence (borrowing some terminology from the debate over Frankfurt‐style cases). If we want to assess whether Jim possessed responsibility‐level freedom with regard to his lying, we must hold fixed the nonoccurrence of the lightning strike, just as we hold fixed nonintervention by a Frankfurt‐style intervener. It is false that in the relevant nearby possible worlds (those in which the lightning strike failed to occur), Jim would not have lied. So the modal account gets this case right.

Hartman may have ruled out limiting the set of possible worlds relevant to assessing the case to those in which the lightning strike fails to occur, on the grounds that such a restriction would make no sense in the context of assessing circumstantial luck. Restricting ourselves to the set of worlds in which the lightning strike does not occur is restricting ourselves to those with (broadly) the same circumstances, and eliminates the possibility of circumstantial luck. That's quite right. If we want to know whether an agent exercised control over their ϕ‐ing, we hold circumstances fixed. That's a lesson that John Martin Fischer taught us; we hold fixed the actual circumstances in order to assess whether agents possess the reasons responsiveness that is at the heart of control (see Fischer 2002; Fischer and Ravizza 1998). A different set of counterfactuals are relevant to assessing whether an agent was subject to circumstantial luck: the relevant counterfactuals are those in which the circumstances vary, in ways that are realistic (roughly, the relevant set of worlds is the set in which the agent might easily have found herself, had things in her life gone somewhat differently; had she taken *that* job rather than *this*; had she gone to *that* school; had her parents emigrated as they were considering, and so on). Yet another set of possible worlds is relevant when we ask about constitutive luck.2Hartman's second objection to views like mine—that they cannot adequately distinguish between luck and fortune—also seems to fail because it does not recognize the way in which my view (at least) shifts between sets of possible worlds in assessing whether something is lucky. The objection goes like this: call a disposition “fortunate” for the agent if it is significant for the agent and she lacks control over it, but it is modally stable. Character traits may be fortunate in this way. Now, such dispositions generate a paradox akin to that generated by moral luck cases, Hartman plausibly claims: neither blaming agents for such traits (when negative) nor excusing them sits easily with us. If that's right, though, then the modal account does not help to explain the paradox—since the traits are modally stable. It should be obvious how I would reply. A trait can be modally stable relative to one set of possible worlds but modally fragile relative to another, depending on what kind of luck is at issue. The set of traits that are modally stable relative to all relevant possible worlds (relevance is sometimes context‐sensitive, but the broadest set of relevant worlds is the set of worlds in human experience plus those accessible from those worlds) is the set of traits that are not lucky at all. In any case, restricting counterfactuals to those in which the lightning strike doesn't occur gets us the result that Hartman suggests—that Jim exercised control over his lying—but accords with the conclusions of the modal view. It is because Jim exercised control over his lying, and therefore he lies in the relevant set of worlds, that we have the intuition that he is responsible for his lying. If he fails to be responsible, it will be for some other reason: his circumstantial luck, for example.

I am therefore not shaken in my conviction that an account of moral luck requires an account of luck, and that the best account will incorporate a modal condition. However, Hartman has another line of attack against views like mine: he claims that it is vulnerable to a variety of counterexamples. Hartman identifies three kinds. I will focus on the first kind, which he apparently takes to be the most challenging, since he concentrates on it and because it is most important for the purposes of this paper. First, however, brief comments on the other two.

The second kind of counterexample involves actions settled by agents’ endowment—that is, the set of traits constitutive of agents—but which are not lucky. Hartman gives the following example: Jeff holds the door open for Sidney, whose hands are occupied by the boxes she is carrying. Jeff's action is not presently lucky, because it issues from his endowment (it is therefore not chancy—he would have acted as he did in most nearby possible worlds). But it is not constitutively lucky, because he does not regard it as significant and because it issues from common dispositions. To be honest, I'm not sure what claim of mine this is a counterexample to. I claim that luck undermines our responsibility for all our actions. I do not claim that all our actions are subject to responsibility‐undermining luck. Jeff does not seem to be due any praise for his action, on the assumption that Hartman is right in claiming that his action issued from common and stable dispositions. If a sufficient proportion of people would have done as Jeff did, then he deserves no praise for his action. If, on the contrary, it took effort to cultivate the disposition or to act on it, then it is unlikely that the disposition is sufficiently common, and it is subject to constitutive luck after all. While I will not discuss it here, similar thoughts seem to apply to Hartman's third kind of counterexample, involving actions that are *not* expressions of agents’ endowments but are modally stable and insignificant.

The first kind of counterexample is the one that Hartman emphasizes. It involves agents whose actions are settled by their endowment (or modified endowment; that is, the set of traits constitutive of them as modified by their own prior acts and omissions) but over which she exercises direct control. Hartman gives the following example: Jane grew up in a family of thieves and has moved into the trade herself. She endorses being a thief. She notices that a neighbor has left his wallet where she can get it, and she decides to steal it. Her so doing is settled by her modified endowment (there are few nearby possible worlds in which she would do otherwise) but she possesses direct control over her actions. Indeed, Hartman thinks that cases like this can be constructed at will.

Hartman thinks that I am committed to denying that agents like Jane lack direct or responsibility‐level control. But as he points out, in *Hard Luck* there is “dearth of argument for the claim that an agent lacks direct control over an event that is settled by her endowment, her modified endowment, or neither.” (49). That's quite right: I do not offer any argument to show that agents like Jane lack direct control. The claim that the luck that features in moral luck cases is lack of control is, after all, a view I reject. My claim is that agents like Jane are not responsible because their endowment (which, we agree, settles how she acts) is lucky for her, not because she lacks control over her action. More generally, luck undermines moral responsibility. One way it does so is by undermining control (when actions are undetermined or stochastic, for example). But luck undermines responsibility in other ways too: for instance, when agents are lucky to be the way they are, and how they are settles how they act.

Perhaps Hartman thinks I am committed to thinking that luck undermines control because I build a control condition into my definition of luck. But that condition requires that the agent lacks control over whatever it is that is lucky for her; not (necessarily) over her actions. Constitutive and circumstantial luck do not reduce our control over our actions; they threaten our responsibility in other ways. It is noteworthy that our tests for constitutive and circumstantial luck involve a different set of counterfactuals than tests for typical cases of resultant luck; we hold fixed the actual circumstances, as Fischer taught us, when we want to assess whether an agent possesses control over her actions. It follows that when we allow the actual circumstances to vary across the counterfactuals, we are not in the business of assessing whether the agent possesses such control.

Hartman has a number of other arguments against my view, which I lack the space to respond to properly here. I will briefly note several of the more powerful, together with the beginnings of a reply.

- In response to my argument that luck significantly reduces our direct control, he notes that agents are often held responsible for actions in virtue of the exercise of indirect control (52). But agents exercise *indirect* control by way of exercising *direct* control. Hartman simply stipulates that the agents in his example exercise responsibility‐level direct control over the actions in virtue of which they exercise indirect control, thereby begging the question against the very view he aims to rebut.
- He argues that anyone who claims (as I do) to be a compatibilist should accept that we have sufficient control over our actions in spite of luck, since they accept that the “great” diminishment of control intrinsic to causal determinism does not undermine control sufficiently to undermine responsibility (54). I think this is a mistake: causal determinism does not undermine control *at all*. In any case, this argument changes the subject: my aim is not to show that luck undermines control and thereby moral responsibility. Rather, my aim is to show that luck undermines moral responsibility directly.
- Hartman claims if determinism is true, whatever factors that are supposed to undermine responsibility through the workings of constitutive and present luck are a proper part of the causally deterministic process. But if causal determinism does not undermine moral responsibility, then no proper part of the deterministic process can undermine responsibility (54). This seems to me to be a bad mistake. If the argument succeeded, it would entail that nothing can undermine moral responsibility if compatibilism is true, since any putative undermining factor (say psychosis) would be a proper part of the deterministic process. The argument also overgeneralizes unacceptably. Consider a skeptical analog in the epistemic domain. Some philosophers argue that since we cannot rule out being brains‐in‐vats, we cannot justifiably claim to know that any *a posteriori* proposition is true. It is surely no response to these philosophers to respond that determinism does not entail skepticism, but if determinism is true then our being brains‐in‐vats is merely a proper part of the deterministic causal process.

Finally, perhaps Hartman thinks I am committed to holding that luck undermines control because while it is easy to see how lack of control can undermine responsibility, it is not at all apparent how the significance or modal fragility conditions could undermine responsibility (51). I agree; lack of control is a threat to moral responsibility and the other conditions do not seem to be. My claim, however, is not that luck undermines responsibility by undermining control: rather it does so directly. I confess I have no response to the question what it is about luck that undermines responsibility other than to point to clear cases and common intuitions. I suspect that any deeper response to this question would consist in showing how the claim coheres with other related concepts and practices. I don't think we can do any better with regard to the question “why does lack of control undermine moral responsibility?” Here we hit ground level. It is precisely because we must appeal to intuition in normative domains like this that it is so important to appeal to the broadest set of considerations we can.3Hartman himself appeals to debates over epistemic luck in constructing his case for the claim that luck can affect responsibility. But epistemic luck is not best understood as turning on lack of control. Consider fake barn cases. The reason that the agent fails to have knowledge in such cases is not that she lacks control, but the modal fragility of her belief being true: in a large proportion of nearby possible worlds, she would not have been standing in front of the one genuine barn in fake barn country (see Levy 2014 for discussion). I now turn to such broader considerations.

## 2 The Politics of Luck

Because what is at issue in the debate over moral luck is, precisely, luck—the same concept that arises in many other debates, within philosophy and beyond it—I don't think we can effectively partition the moral luck debate from these other questions. What we conclude about moral luck will have implications far beyond the kinds of questions that the debate tends to focus on. It will have such implications because answers to questions about moral luck will entail, or at least make more plausible, particular responses to other problems and particular takes on other issues. Questions concerning luck play a particularly obtrusive role in political life, and the views we come to regarding moral luck have implications for how we should think of luck when it comes to distributive justice.

We may make sense of central debates between left and right in contemporary democracies as debates that turn in very important part on the existence and significance of luck. Those on the left advocate redistributive taxation to compensate those who are the victims of bad luck.4It is noteworthy, however, that the correlation between the acceptance of luck as an important factor in determining outcomes and being on the left politically is much weaker in the US than in Europe. Americans, left and right, are much less accepting of a role for luck than Europeans (Alesina, Sacerdote, and Glaeser 2001). A central justification for redistributive policies is that those who contribute to such schemes do not *deserve* (all of) their high income, because it is the fruit (in part) of luck, while those who are unemployed, or in ill‐health do not deserve their fate, because it too is (in part) the product of luck. Of course, it is entirely possible to want to improve the condition of the needy without thinking that they are the victims of bad luck, or that the difference between them and those who are better off is (very significantly) luck. Indeed, many people on the right happily donate to charities. But it is harder to motivate compulsory redistribution if we believe that those who are better off deserve their wealth.

It is not controversial that as a matter of fact there is a correlation between the political right and a denial of the significance of luck. Witness the furore that greeted Obama's (in)famous “you didn't build that” statement. Obama noted that successful business people did not “build” the education system that prepared them, the roads their goods travel on, the infrastructure they use, and so on. Mitt Romney called the statement “insulting” and conservative commentators called it an attack on personal responsibility.5Wikipedia has a good summary of the context and aftermath of Obama's speech. <https://en.wikipedia.org/wiki/You_didn't_build_that#Romney_campaign>. There are also more systematic data demonstrating the correlation between right‐wing views and the denial of luck. Dena Gromet, Kimberly Hartson, and David Sherman (2015) found that conservatives were unwilling to attribute success to luck, except to the extent to which they saw luck as a property of the individual, and Jasmine Carey and Delroy Paulhus (2013) found that conservatives both had lower rates of belief in luck and accepted bad luck as an excuse to a lesser extent than liberals. There is also evidence that acceptance that luck plays a role in outcomes correlates with belief in the need for a robust welfare system, with differences in the degree to which citizens of countries hold that poverty is significantly due to luck correlating with the extent of the social safety set (Alesina, Sacerdote, and Glaeser 2001).

It is very difficult to see how it is possible entirely to insulate the moral luck debate from these political issues. Those on the right believe that the wealthy (often) deserve their riches, and if luck can affect desert, they may be right. After all, wealth very often arises from some combination of precisely the same kinds of luck that are supposed to affect moral desert. Intelligence correlates quite strongly with income (Zagorsky 2007), and intelligence is the product of constitutive luck (luck in genes, in formative environment, in feedback from others). Intelligence actually predicts income much better than it predicts wealth, but that's no comfort for those who think that wealth is earned. Very great wealth is better predicted by having extremely wealthy parents (either because very great wealth is simply inherited or because the stakes needed for acquisition of very great wealth are inherited), and chance—present luck—explains much of the remaining variance, at least among those whose wealth stems from investing (Levy and Levy 2003).

There is a widespread view, defended by Daniel Dennett (2015) among others, that luck tends to even out, so it can be safely ignored. If life were a 100‐yard dash, Dennett writes, then it would be unfair to give some a head start. But life is a marathon, not a sprint. In fact, Dennett points out, the best marathon runners are seeded and as a result placed in the front group, but that's not unfair because the race is a long one, offering plenty of opportunities for the luck to even out. But luck does not tend to even out, and marathon seeding is a perfect illustration of this fact. Because they are seeded in the front group, the fastest runners do not have to fight their way through the congestion of other, slower, runners. They are, of course, the most talented and best trained runners; the ones who least need an extra advantage: they get one nevertheless.

Of course, athletics represents an artificial environment and various kinds of handicaps and advantages may be unobjectionable. But luck ramifies, rather than canceling out, in ordinary life. There are a variety of positive feedback loops which ensure that small initial advantages tend to grow over time, not diminish (Frank 2016). Consider, for example, two children, one of whom has a small initial advantage in a capacity that happens to be valued by those around her (say running). This small advantage will tend to be rewarded, because it allows the better performing child to do better in competition; it will thereby be reinforced. Her talent may be identified by adults who encourage her and may coach her. In the right environment, she will receive rewards that are extrinsic and intrinsic (extrinsic: medals; praise; intrinsic: the joys of running well). The small initial advantage may, over the course of a decade, transform into a gulf between her and other children. It may be true that had the initially less talented child received the same feedback, he might have done just as well or even better. But because he did not, he languishes in the back of the pack. This kind of effect is, once again, backed by data. In professional hockey, roughly 40 percent of all players were born in the first 3 months of the year while only 10 percent were born in the last 3 months. This disparity is explained by the fact that January 1 is used as the cut‐off date for participation in age‐grouped youth hockey leagues. As a result, the sooner one's birth date after January 1, the bigger and stronger one will be, on average, relative to one's age cohort. Of course, the initial difference is small, and since professional hockey is not age‐grouped by itself it would have no effect on who goes onto the big leagues. But because it is reinforced and thereby grows, the small initial difference translates into different life trajectories (Frank 2016).

The ways in which initial differences in constitutive luck ramify would matter much less if small initial advantages were evenly distributed. But they are not: initial advantages in valued skills tend to cluster. Better nutrition, for example, predicts higher IQ, greater fitness, and a greater capacity to attend to school lessons (Adolphus et al. 2016). As a result, those lucky to be born into better resourced families are more likely to manifest the small initial advantages which may grow into gulfs. Advantage is *systematic* and *reinforced* by positive feedback loops. At the same time, those with few initial advantages may be subject to negative feedback loops. By the time they are in middle school, for example, they may find school work difficult and frustrating, and therefore tend not to try as hard.

Those who embrace moral luck may respond that I have simply changed the subject. The inequalities that characterize our societies may be a matter of urgent concern, but they are irrelevant to the moral luck debate. The question whether those who have the good fortune to have marketable skills, persistence, and a capacity for hard work deserve the wealth they accrue is entirely dissociable from the question whether the agent who drives recklessly deserves more blame if she hits a pedestrian than another agent who, by luck, did not. At most, the defender of moral luck might concede that there is a psychological tendency to conflate them, but hold that this is a mistake that can and should be resisted.

I doubt, however, that we can segregate the questions in this way. The same processes that lead to advantages and disadvantages with regard to talents lead, in precisely the same kinds of ways, to the acquisition of dispositions that are morally assessable (such as dispositions toward criminality). They are equally the product of constitutive and circumstantial luck, and they correlate with one another (the capacity to resist temptation, to take a central example, is correlated with socioeconomic status; Hackman, Farah, and Meaney 2010).6Of course, those with better resources may be less tempted by criminal acts in the first place, because they know that they have available to them strategies for securing rewards that are better bets than theft. Once again, we see how advantage, on the one hand, and disadvantage, on the other, ramify: those who are better able to resist temptation are also less likely to be subject to it, and vice versa. The same kind of present luck that distinguishes one reckless driver from another is also at work in explaining the differences between those whose risky investments pay off and those whose did not. In Robert Frank's (2016) simulations, which held talent and effort constant, in only a small minority of cases did the rewards of winner‐take‐all competitions go to the most talented and hardworking. Why is one set of rewards and punishments deserved and not the other, given that they are explained by the same processes, and given the considerable overlap between them?

Indeed, there is a case for holding that the overlap between the realm of distributive justice and the domain of moral luck is considerable: on some views, the political domain is the moral realm writ large. It is common to object to those doctrines according to which wide disparities in wealth are tolerable because they are independent of the kind of equality that matters morally—equality before the law, equality of dignity, equality of rights and liberties—strip egalitarianism of its moral content. It is this kind of formal equality that Anatole France mocked when he spoke of the majestic equality of the law, which forbids rich and poor alike from sleeping under bridges or stealing loaves of bread. Formal equality of opportunity certainly obscures substantive inequality of opportunity: jobs may be open to talents, but talents are not distributed equally: they are nurtured and inculcated in those who least need them, because they will inherit opportunities and resources not open to those who are not in line for any of this largesse.

Even if we restrict our attention to the paradigm cases of moral luck, these political issues lurk in the background. A number of philosophers of biology have proposed replacing the notion of innateness, which is of little use in biology, with canalization, where a trait is canalized if it is buffered against developmental perturbation. Buffering keeps trait development on track: through the use of redundancy and feedback mechanisms, external interference with development is compensated for. Buffering is a way of minimizing the effects of chancy luck. Some agents are buffered against luck, and this buffering is systematically distributed. We might understand “privilege,” at least in one of its dimensions, as consisting in such buffering.7Rachel McKinnon (2014) argues that skill increases the opportunities for an agent to be lucky. I suspect that's true only in a limited range of cases—those, like the ones she focuses on, in which the skilled agent is more likely to stay in the game longer than the less skilled—and it also obscures the more important ways in which skill minimizes luck, rather than increasing it. The skilled agent needs less luck to be successful: the proportion of possible worlds in which she fails decreases as her skill increases. In Levy (2011) I argued that planning, too, minimizes luck. Buffering against luck should be understood along similar lines: it provides the pathways to ensure that chance deviations are unlikely to disrupt the developmental path. It is such buffering that parents deliberately aim to provide to their children: a good education, good connections, cultural capital, and money all ensure that bad luck is less likely to be catastrophic. Social mobility statistics provide a powerful demonstration that buffering against luck works. The job market, for example, is systematically less risky for some agents than for others: if they miss out on their first jobs, their second choices are pretty good too (whether this is ensured through family connections, a good education, or having the right accent). Even outcomes differ across cases: two agents driving equally riskily have different probabilities of causing a harm, if one is driving a more responsive car than the other, and if the harms eventuate, the better resourced agent may be in a position to ensure that he is in a position to repair it, by paying for rebuilding or medical care. Since of course we rarely actually compare pairs of cases with all relevant details held constant in the real world, holding agents responsible for the harms they cause will tend in the actual world to favor better resourced agents over less well resourced.8Note that buffering against luck reduces susceptibility to some kinds of luck; it does not eliminate the decisive influence of luck. Buffering reduces the influence of present or—sometimes—circumstantial luck, but it is itself the product of circumstantial or constitutive luck. One is lucky to be well buffered.


None of this constitutes anything like a knockdown argument against the defenders of moral luck, of course. They may respond that they are concerned to show that in possible worlds in which everything is held constant, causing a worse harm is grounds for more blameworthiness, and point out that things are not held constant in the actual world, because different agents have very different starting points (captured by my talk of buffering against luck). I am skeptical that the defender of moral luck can make this response work for all kinds of luck, since it seems to require denying constitutive and circumstantial luck in order to defend resultant (and it would obviously dramatically limit the real world implications, even for resultant luck). Alternatively, they may simply deny that the injustices to which I've pointed are genuine injustices at all. They may hold that systematically distributed differences in luck across individuals are fair, or at any rate not unfair. Finally, they may attempt to restrict their defense of luck to the kinds of cases that have been the mainstay of the moral luck debate, and deny that they are committed to holding that luck in other domains is fair. Taking that tack would seem to require them to show either that the luck at issue is of a different kind, or that it has different normative implications in one domain than another; I can't see how the argument would go.9It is noteworthy that at least some defenders of luck accept that their view does have more general implications. Hartman suggests that reflection on moral luck produces the understanding that “every person is significantly shaped by constitutive, circumstantial, and resultant luck. This belief may help one to avoid an attitude of superiority and a deficit of compassion. It may also promote moral humility” (14). Such reflection “may promote a certain kind of generosity, charity, and patience with others—especially with those who have not been the beneficiaries of such good luck” (15). If Hartman is right that moral luck can affect desert, however, then at very minimum the extent to which attitudes of superiority are combatted and compassion promoted by reflection on moral luck seems to be dramatically reduced. If moral luck cannot affect desert, then such reflection should lead us to think that I do not deserve the wealth, the health, the regard of others or even the freedom I have, any more than someone else who lacks one or more of these things does. That should indeed induce humility in me and compassion for those who have been unluckier. But if Hartman is right, then reflection on moral luck should lead me to see that I do deserve my freedom and the regard of others, even though I would not have these things had I been less lucky. Since I deserve the benefits and others do not, why should I be any more compassionate that I was before reflection? After all, *by hypothesis* I do not think that the influence of moral luck should lead to less punishment for those who have been unlucky. What kind of compassion is this, which does not find expression in thinking that punishment should not be reduced?


My aim in this second section has not been to show that none of these options will work. Rather, I am more concerned with setting out the issues that are at stake. A defender of moral luck needs to consider the implications of their view for these wider questions. These issues therefore serve as additional constraints on the acceptability of their views: they must show that, and how, they are able to avoid taking a position on these questions (whether by showing that the “luck” in the “moral luck” debate is a different kind, or by showing how the issues differ) or they must defend their view. We like to proceed by imagining idealized cases and pumping intuitions, but unless we're prepared to accept that our conclusions apply only in possible worlds very unlike this one, we need also to think how our cases play out in the actual world. Doing so is both important in its own right and productive, insofar as more constraints better enable the defense of a unique option.

I have argued we need to put the “luck” back into moral luck. It is luck itself, and not a pale shadow, that is at issue. Lack of control by itself does not allow us to understand the traditional moral luck cases adequately. But restoring luck opens the debate onto wider issues. I have sketched a small range of these wider issues, which I take to be worth sketching due to their intrinsic importance and their neglect hitherto. An adequate account of luck will attend to many more (especially to the rich experimental work on luck intuitions).10In Levy (2016), I offered an evolutionary account of resultant luck intuitions, which I suggested can play a role in an overall argument for rejecting its moral significance. Hartman suggests that he can ignore work like this, because an argument for the reality of moral luck entails the falsity of error theories like mine. This is a methodologically inapt response, given that Hartman's positive account relies heavily on intuitions. Insofar as error theories offer a debunking account of those very intuitions, reliance on them requires a response to the error theory. We must take luck seriously, theoretically and practically. That is, we must be alive to the best work on luck, wherever we find it, and we must bear in mind the significance of luck in our actual lives as moral and political beings.11This work is supported by a generous grant from the Wellcome Trust (WT104848/Z/14/Z). I am grateful to Gregg Caruso for sharing his own paper responding to Hartman with me; that paper stimulated some of the points made here. I am also grateful to Andrew Khoury for helpful comments on a draft of this paper.

